# Glyoxalase 1 gene is highly expressed in basal-like human breast cancers and contributes to survival of ALDH1-positive breast cancer stem cells

**DOI:** 10.18632/oncotarget.26369

**Published:** 2018-11-23

**Authors:** Shoma Tamori, Yuka Nozaki, Hitomi Motomura, Hiromi Nakane, Reika Katayama, Chotaro Onaga, Eriko Kikuchi, Nami Shimada, Yuhei Suzuki, Mei Noike, Yasushi Hara, Keiko Sato, Tsugumichi Sato, Kouji Yamamoto, Takehisa Hanawa, Misa Imai, Ryo Abe, Atsushi Yoshimori, Ryoko Takasawa, Sei-Ichi Tanuma, Kazunori Akimoto

**Affiliations:** ^1^ Department of Medicinal and Life Science, Faculty of Pharmaceutical Sciences, Tokyo University of Science, Chiba, Japan; ^2^ Department of Pharmacy, Faculty of Pharmaceutical Sciences, Tokyo University of Science, Chiba, Japan; ^3^ Research Institute for Biochemical Sciences, Tokyo University of Science, Chiba, Japan; ^4^ Department of Information Sciences, Faculty of Science and Technology, Tokyo University of Science, Chiba, Japan; ^5^ Department of Biostatistics, Yokohama City University, School of Medicine, Yokohama, Japan; ^6^ Translational Research Center, Research Institute for Science and Technology, Tokyo University of Science, Chiba, Japan; ^7^ Laboratory of Genomic Medicinal Science, Research Institute for Science and Technology, Tokyo University of Science, Chiba, Japan; ^8^ Department of Hematology, Juntendo University School of Medicine, Tokyo, Japan; ^9^ Leading Center for the Development and Research of Cancer Medicine, Juntendo University School of Medicine, Tokyo, Japan; ^10^ Institute for Theoretical Medicine, Inc., Kanagawa, Japan; ^11^ Strategic Innovation and Research Center, Teikyo University, Tokyo, Japan

**Keywords:** cancer stem cell, glyoxalase 1, breast cancer, the Warburg effect, target therapy

## Abstract

Glyoxalase 1 (GLO1) is a ubiquitous enzyme involved in the detoxification of methylglyoxal, a cytotoxic byproduct of glycolysis that induces apoptosis. In this study, we found that GLO1 gene expression correlates with neoplasm histologic grade (*χ*^2^ *test*, *p* = 0.002) and is elevated in human basal-like breast cancer tissues. Approximately 90% of basal-like cancers were grade 3 tumors highly expressing both *GLO1* and the cancer stem cell marker *ALDH1A3*. ALDH1^high^ cells derived from the MDA-MB 157 and MDA-MB 468 human basal-like breast cancer cell lines showed elevated GLO1 activity. GLO1 inhibition using TLSC702 suppressed ALDH1^high^ cell viability as well as the formation of tumor-spheres by ALDH1^high^ cells. GLO1 knockdown using specific siRNAs also suppressed ALDH1^high^ cell viability, and both TLSC702 and GLO1 siRNA induced apoptosis in ALDH1^high^ cells. These results suggest GLO1 is essential for the survival of ALDH1-positive breast cancer stem cells. We therefore conclude that GLO1 is a potential therapeutic target for treatment of basal-like breast cancers.

## INTRODUCTION

Breast cancer is the most commonly occurring cancer in women worldwide, with 2.5 million new cases (30% of all cancers in women) and 0.4 million cancer-related deaths (14% of all cancer deaths in women) reported in 2017 [1]. Based on its receptor status, breast cancer is traditionally categorized as ER-positive, PgR-positive, HER2-positive, or triple-negative (ER-negative, PgR-negative, HER2-negative) (TNBC). Breast cancer is also classified into subtypes distinguished based on differences in their gene expression patterns (PAM50), including normal-like, luminal A, luminal B, HER2-enriched, claudin-low and basal-like [2–4]. Among these, 70–80% of basal-like breast cancers reportedly fall into TNBC category [5]. Basal-like breast cancers have stem-like properties and a poor prognosis [6]. There are currently no treatment options other than conventional surgery, chemotherapy and radiotherapy available to these patients. Identification and development of novel therapeutic targets for basal-like tumors are therefore much needed.

Cancer stem cells (CSCs) are a small subpopulation of cancer cells exhibiting a capacity for self-renewal, multipotency and tumorigenesis; consequently, CSCs are thought to be the main drivers of tumorigenesis [7, 8]. CSCs also serve as the seed for tumor recurrence after medical treatment, as most CSCs are resistant to several standard antitumor treatments, including both chemotherapy and radiotherapy. However, a detailed understanding of the mechanisms that define the properties of CSCs could potentially contribute to detection of novel therapeutic targets and drugs.

CSCs in breast cancer patients can be identified based on expression of such marker molecules as CD44^high^/CD24^–/low^ and aldehyde dehydrogenase 1 (ALDH1) [9]. ALDH1, an enzyme that converts aldehydes to carboxylic acids, is abundant in normal stem/progenitor cells and also exhibits higher activity in various epithelial CSCs, including breast cancer [10, 11]. Moreover, several studies have shown that two isoforms, ALDH1A1 and ALDH1A3, are useful markers for isolating and tracking human CSCs [12–14]. In breast cancer, ALDH1A3 reportedly contributes significantly to ALDH1 activity, and its expression correlates significantly with cancer type, tumor grade and metastasis [15, 16].

Glyoxalase 1 (GLO1) is a key cytoprotective enzyme functionally linked to methylglyoxal (MG) degradation [17]. MG is a cytotoxic glycolysis byproduct that is highly reactive with DNA/RNA and proteins, and induces apoptosis in tumor cells [18]. Moreover, high GLO1 expression has been observed in a variety of human cancers, including leukemia [19] and cancers of the lung [20], stomach [21, 22], colon [23], pancreas [24], liver [25, 26], prostate [27, 28], oropharynx [29], skin [30, 31] and breast [32]. In addition, GLO1 activity is reportedly elevated in many cancer types [19, 20]. Interestingly, analysis of a small number of specimens suggests that among breast cancer subtypes, GLO1 activity is highest in TNBC [33]. In Bcr-Abl^+^ leukemia, hypoxia-adapted (HA)-Bcr-Abl^+^ cells exhibiting stem cell-like characteristics showed higher GLO1 expression and enzymatic activity, and GLO1 inhibitors effectively suppressed the viability and capacity for tumor formation of this HA-Bcr-Abl^+^ cells [34]. However, the role of GLO1 in ALDH1-positive CSCs in basal-like tumors remains unclear. We therefore investigated GLO1 expression in breast cancer subtypes and its function in ALDH1-positive CSCs. Our findings suggest GLO1 is a potentially useful therapeutic target in ALDH1-positive CSCs in basal-like tumors.

## RESULTS

### GLO1 gene (*Glo1*) is highly expressed in grade 3 breast tumors with low frequency of gene amplification

**Figure 1:**
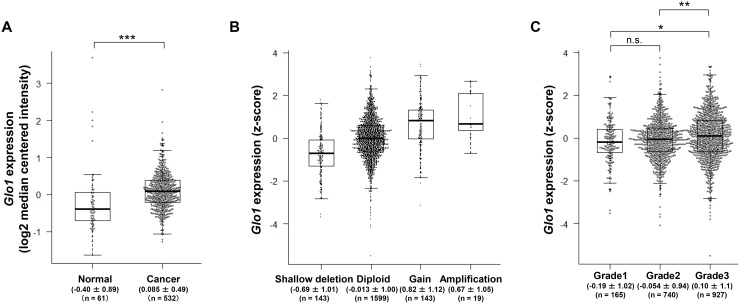
*Glo1* is overexpressed in grade 3 breast cancer tumors despite a low frequency of gene copy number alternation and genetic mutation (**A**) Box plot comparing *Glo1* expression in normal (*n* = 61) and cancer tissue (*n* = 532) (from the TCGA dataset). Values indicate the medians ± SD. ^***^p < 0.001; Mann-Whitney *U* test. (**B**) Effect of copy number status on *Glo1* expression: shallow deletion (*n* = 143), diploid (*n* = 1599), gain (*n* = 143), and amplification (*n* = 19) (from the METABRIC dataset). Values indicate the medians ± SD. (**C**) *Glo1* expression in grade 1 (*n* = 165), grade 2 (*n* = 740), and grade 3 (*n* = 927) tumors (from the METABRIC dataset). Values indicate the medians ± SD. ^*^p < 0.05, ^**^p < 0.01, n.s. = not significant; Kruskal-Wallis test with Steel-Dwass test. Center line, median; box limits, upper and lower quartiles; whiskers, ± 1.5 × IQR; points, all data points.

To examine *Glo1* expression and mutation in breast cancer, we used two different datasets: the TCGA dataset, which also includes data from normal tissues, and the METABRIC dataset, which lacks data from normal tissues. We initially compared *Glo1* expression in normal (*n* = 61) and cancer tissues (*n* = 532) from breast cancer patients using the TCGA dataset. As shown in Figure 1A, *Glo1* expression was higher in the cancer tissues than normal tissues (Mann-Whitney *U* test, *p* < 0.001). Because *Glo1* amplification often occurs in gastric [22] and liver cancers [25], we examined the alterations in copy number and mutations in *Glo1* in breast cancer tissue. We found that the frequency of *Glo1* copy number alteration was low, and amplification of the gene was observed in only 19 of 1904 (1.0%) patients (Figure 1B). In addition, no genetic mutations, including missense, in-frame and truncation, occurred in *Glo1* (0 of 1904).

We next examined in detail the relationship between *Glo1* expression and the clinicopathological data in the TCGA and METABRIC datasets. There was no correlation between *Glo1* expression and the clinicopathological data in TCGA dataset (Table 1). On the other hand, *Glo1* expression correlated with Neoplasm histologic grade in the METABRIC dataset (Table 2, χ^2^ test, *p* = 0.002). In addition, *Glo1* expression was significantly higher in Grade 3 tumors than in Grade 1 or 2 tumors (Figure 1C, Steel-Dwass test, Grade 1 vs. Grade 3: *p* = 0.014, Grade 2 vs. Grade 3: *p* = 0.005). This is consistent with the earlier report that GLO1 expression at protein level correlates with tumor grade in breast cancer specimen [35]. These results indicate that GLO1 overexpression with the low frequency of gene amplification and no genetic mutations may play important roles in Grade 3 tumors and in cancerous progression.

**Table 1:** χ^2^ test of the association between clinicopathologic parameters and *Glo1* expression using the TCGA dataset

Clinical and pathological factor		*Glo1*^low^	*Glo1*^high^	*p* value
Primary tumor				
	T1	36	60	0.18
	T2-4	160	195	
Regional lymph nodes				
	N0	97	119	0.61
	N1-4	96	130	
Distant metastasis				
	M0	189	238	0.057
	M1	2	10	
Tumor stage				
	Stage I-II	125	167	0.053
	Stage III-IV	34	72	
Estrogen receptor status				
	Negative	43	52	0.052
	Positive	93	180	
Progesterone receptor status				
	Negative	54	90	0.83
	Positive	83	145	
ERBB2 status				
	Negative	98	130	0.37
	Positive	27	46	

**Table 2:** χ^2^ test of the association between clinicopathologic parameters and *Glo1* expression using the METABRIC dataset

Clinical and pathological factor		*Glo1*^low^	*Glo1*^high^	*p* value
Tumor size (mm)				
	0–20	307	287	0.44
	≥ 20	643	649	
Tumor stage				
	Stage 0–II	657	622	0.64
	Stage III–IV	61	63	
Neoplasm histologic grade				
	Grade 1–2	489	416	0.002
	Grade 3	435	492	
ER status				
	Negative	242	203	0.067
	Positive	721	738	
PgR status				
	Negative	450	445	0.81
	Positive	513	496	
HER2 status				
	Negative	848	820	0.54
	Positive	115	121	

### *Glo1* is highly expressed in basal-like breast cancer

**Figure 2:**
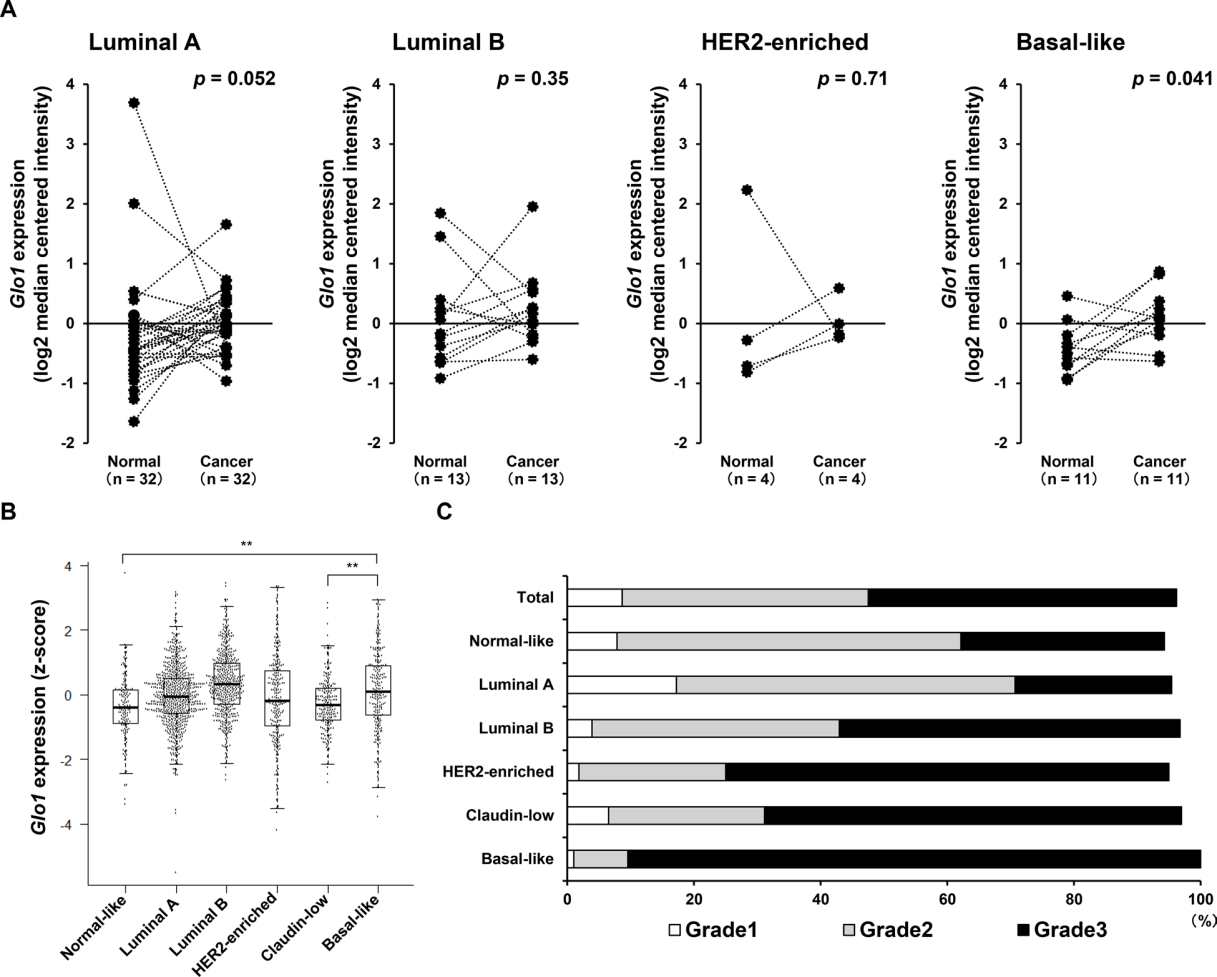
*Glo1* is overexpressed in basal-like breast cancers (**A**) Paired comparison of *Glo1* expression in normal tissue and tumor tissue from each subtypes using TCGA dataset (Wilcoxon signed rank test: Luminal A, *n* = 32 in each group; Luminal B, *n* = 13 in each group; HER2-enriched, *n* = 4 in each group; Basal-like, *n* = 11 in each group) (from the METABRIC dataset). (**B**) *Glo1* expression in breast cancer subtypes: centerline, median; box limits, upper and lower quartiles; whiskers, ± 1.5 × interquartile range (IQR); points, all data points. ^**^p < 0.01; Kruskal-Wallis test with Steel-Dwass test. (**C**) Proportions (%) of tumor grades in each subtype (from the METABRIC dataset).

Comparison of *Glo1* expression in subtypes of breast cancer and normal tissues derived from the same patients in the TCGA dataset revealed that *Glo1* expression was significantly higher in basal-like cancers than normal tissues (Figure 2A). Interestingly, in the METABRIC dataset (*n* = 1904), where *Glo1* was highly expressed in luminal B and basal-like breast cancers (Figure 2B), approximately 90% (180 of 199 patients) of basal-like tumors were classified as neoplasm histologic grade 3 (Figure 2C). These results suggest GLO1 plays an important role in the progression of basal-like cancers.

### GLO1 activity is enhanced in ALDH1^high^ cells isolated from basal-like human breast cancer cell lines

**Figure 3:**
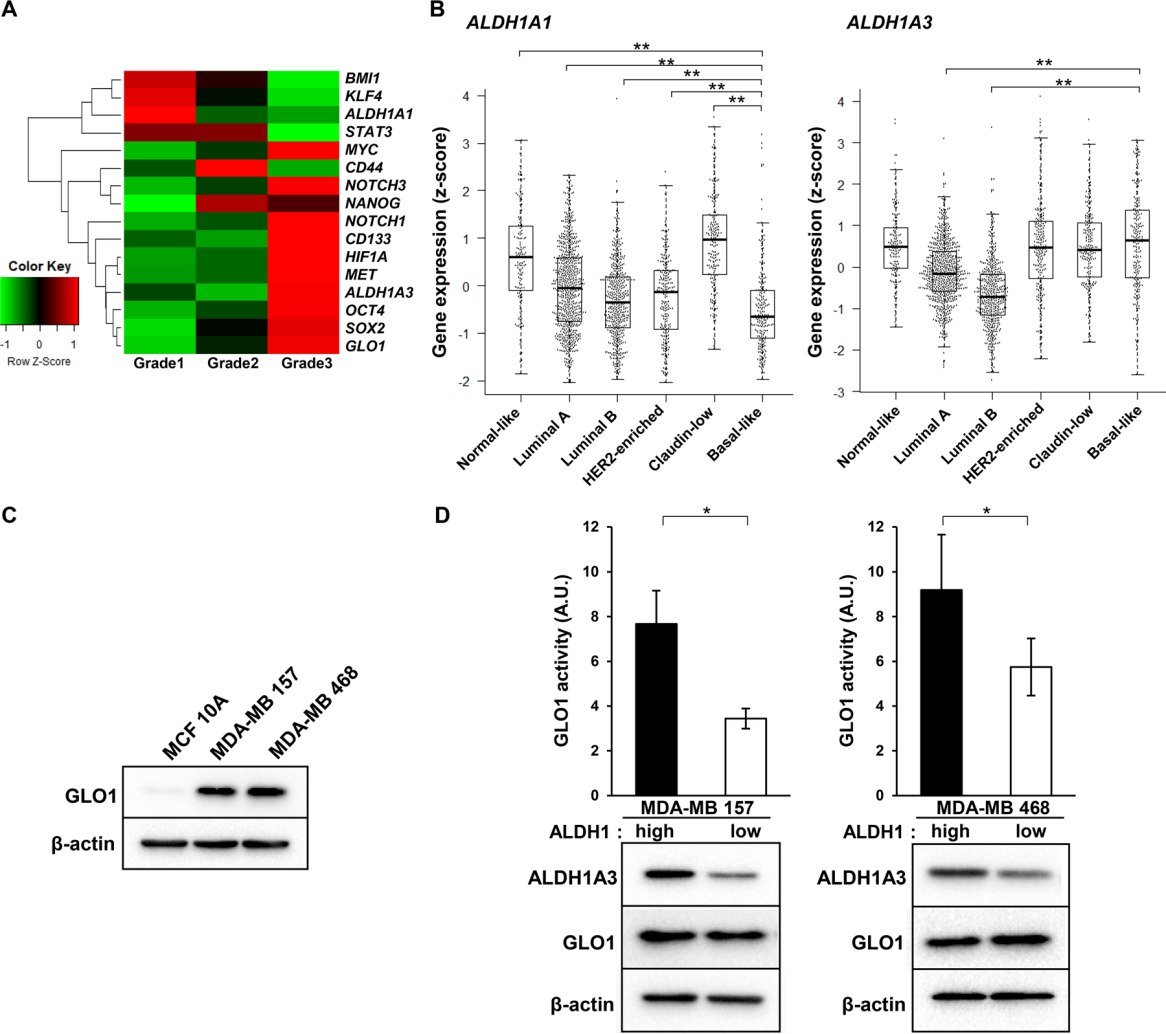
GLO1 activity is enhanced in ALDH1^high^ cells isolated from basal-like breast cancer cell lines (**A**) Heatmap of the average level of stemness gene expression (z-score) in each breast cancer grade (*n* = 1904) (from the METABRIC dataset). Raw z-scores were recalculated based on the average values. In the heatmap, red represents upregulated genes and green represents downregulated genes. (**B**) Box plot comparing *ALDH1A1* (left) and *ALDH1A3* (right) gene expression in each tumor subtype (from the METABRIC dataset): centerline, median; box limits, upper and lower quartiles; whiskers, ± 1.5 × interquartile range (IQR); points, all data points. ^**^p < 0.01; Kruskal-Wallis test with Steel-Dwass test. (**C**) Immunoblot analysis of GLO1 expression in MCF 10A, MDA-MB 157 and MDA-MB 468 cells. β-actin was used as an internal control. (**D**) Top: comparison of GLO1 activity in ALDH1^high^ and ALDH1^low^ cells (6.0 × 10^3^ cells/well) isolated from MDA-MB 157 and MDA-MB 468 cells. Activity is expressed in arbitrary units (A.U.). ^*^p < 0.05; Students *t*-test. Data represent mean ± SE (three independent experiments). Bottom: immunoblot analysis of GLO1 and ALDH1A3 expression in ALDH1^high^ and ALDH1^low^ cells isolated from MDA-MB 157 and MDA-MB 468 cells. β-actin was used as an internal control.

Grade 3 tumors are characterized as undifferentiated and aggressive, with a loss of tubules and high mitotic activity [36]. Basal-like tumors exhibit more stemness characteristics than other breast cancer subtypes [37]. We therefore hypothesized that grade 3 tumors also highly express stem cell marker genes. As expected, in grade 3 tumors, not only *Glo1* but also marker genes for stem cells, including *c-Myc*, *Nanog*, *Notch1/3*, *CD133*, *HIF1A*, *c-Met*, *ALDH1A3*, *Oct4* and *Sox2*, were highly expressed (Figure 3A). Among these, *ALDH1A3* reportedly contributes significantly to ALDH1 activity in breast cancer cells, and its expression correlates significantly with tumor grade in breast tumor patients [38]. In fact, whereas *ALDH1A1* gene expression was lowest in basal-like tumors, *ALDH1A3* expression was enriched in normal-like, claudin-low, HER2-enriched and basal-like tumors (Figure 3B). Among these subtypes, highly expression of both *Glo1* and *ALDH1A3* were observed in basal-like tumors (Figure 2B, 3B). We therefore examined the role of GLO1 in ALDH1-positive CSCs in MDA-MB 157 and MDA-MB 468 human basal-like breast cancer cells, where GLO1 is overexpressed as compared to MCF 10A human normal-like (non-transformed) mammary epithelial cells (Figure 3C). We previously reported that ALDH1^high^, but not ALDH1^low^, MDA-MB 157 and MDA-MB 468 cells exhibit cancer stem cell features [39]. ALDH1A3 levels were higher ALDH1^high^ than ALDH1^low^ cells (Figure 3D), which is consistent with observations in human cholangiocarcinoma cells [40]. Notably, although GLO1 expression was found to be the same in ALDH1^high^ and ALDH1^low^ cells, GLO1 activity was significantly higher in ALDH1^high^ than ALDH1^low^ cells (Figure 3D). These results suggest that GLO1 plays a key important role in ALDH1-positive CSCs in basal-like tumors.

### TLSC702 suppresses tumor-sphere formation by ALDH1^high^ cells

**Figure 4:**
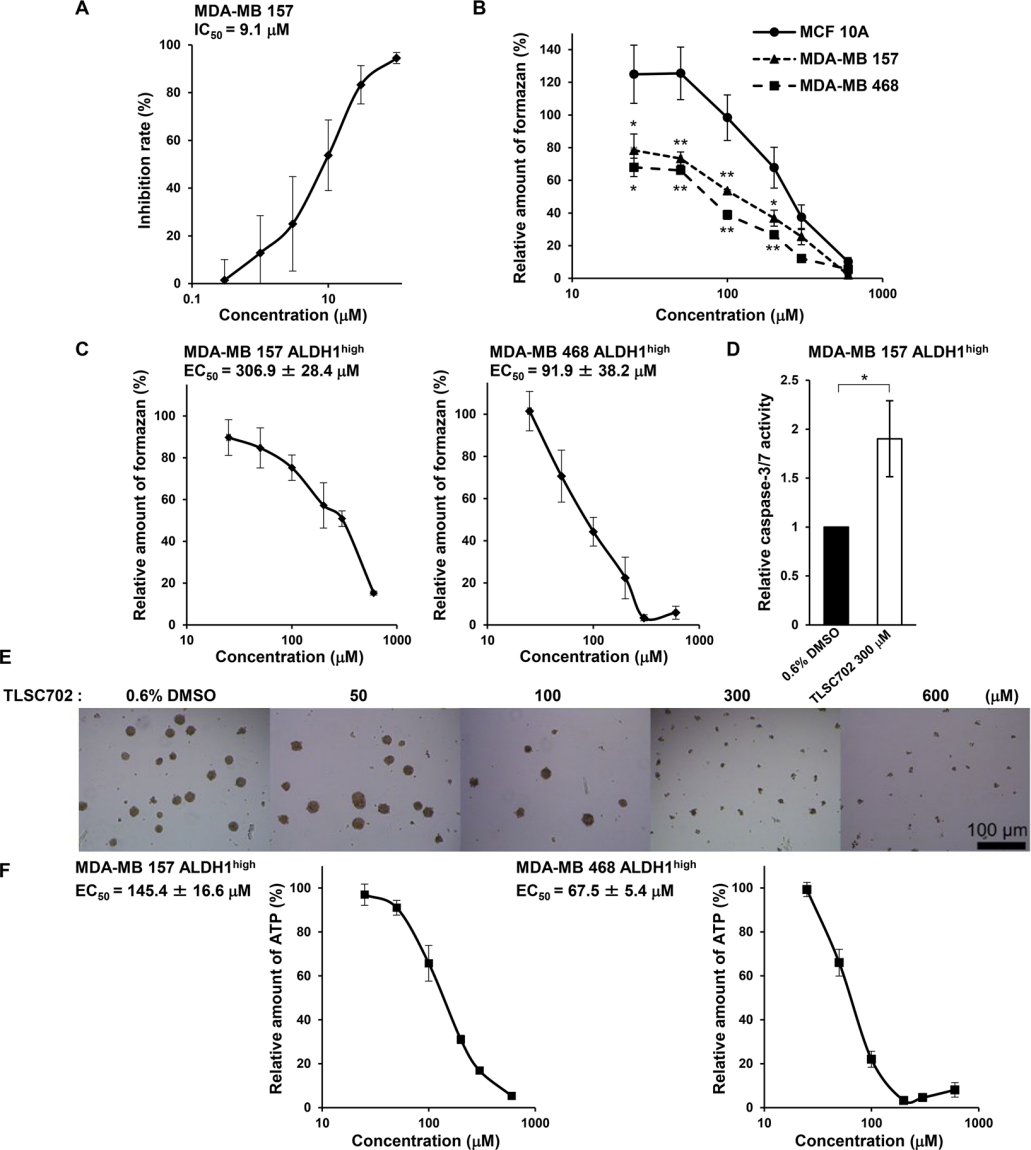
The GLO1 inhibitor TLSC702 induces apoptosis and suppresses tumor-sphere formation by ALDH1^high^ cells (**A**) Inhibition of GLO1 activity in MDA-MB 157 cell extracts by TLSC702. Data represent the mean ± SE (three independent experiments). (**B**) Viability of MCF 10A, MDA-MB 157 and MDA-MB 468 cells treated for 3 days with TLSC702 (25, 50, 100, 200, 300 and 600 μM) was assessed in WST-8 assays. Values for the test groups are expressed relative to cells treated with 0.6% DMSO. Data represent the mean ± SE (three independent experiments). ^*^p < 0.05, ^**^p < 0.01; Dunnett test (MCF 10A vs. MDA-MB 157 or MDA-MB 468 at each concentration). (**C**) Viability of ALDH1^high^ cells isolated from MDA-MB 157 and MDA-MB 468 cells treated for 3 days with TLSC702 (25, 50, 100, 200, 300 and 600 μM) was assessed in WST8 assays. Values for the test groups are expressed relative to cells treated with 0.6% DMSO. Data represent the mean ± SE (three independent experiments). (**D**) Caspase-3/7 activity in ALDH1^high^ cells derived from MDA-MB 157 cells, with and without TLSC702. ^*^p < 0.05; Students *t*-test. Data represent the mean ± SD (three independent experiments). (**E**) Representative images of tumor-spheres by ALDH1^high^ cells isolated from MDA-MB 157 cells treated for 6 days with TLSC702. (**F**) ATP levels in tumor-spheres by ALDH1^high^ cells isolated from MDA-MB 157 and MDA-MB 468 cells treated for 6 days with or without TLSC702 (25, 50, 100, 200, 300 and 600 μM). ATP levels were assessed using Cell-Titer Glo assays. Values for test groups expressed relative to cells treated with 0.6% DMSO. Data represent the mean ± SE (three independent experiments).

To investigate the function of GLO1 in ALDH1-positive CSCs in basal-like tumors, we examined the effects of inhibiting GLO1 activity using TLSC702 [41] in ALDH1^high^ cells isolated from MDA-MB 157 and MDA-MB 468 cells. TLSC702 inhibited GLO1 activity in MDA-MB 157 cell extracts in a concentration-dependent manner, with an IC_50_ of 9.1 μM (Figure 4A). Interestingly, the inhibitory effect of TLSC702 on cell viability was significantly less potent in MCF 10A cells (EC_50_: 249.8 ± 47.8 μM) than in MDA-MB 157 cells (EC_50_: 128.9 ± 23.6 μM) or MDA-MB 468 cells (EC_50_: 79.9 ± 7.6 μM) (Figure 4B). This suggests that basal-like cancer cells are more highly sensitive to GLO1 inhibition than normal mammary epithelial cells.

Upon exposure to TLSC702 in WST-8 cell viability assays, ALDH1^high^ cells exhibited a concentration-dependent decrease in cell viability (Figure 4C). The EC_50_ values were 306.9 ± 28.4 μM (MDA-MB 157) and 91.9 ± 38.2 μM (MDA-MB 468). TLSC702 also enhanced caspase-3/7 activity in MDA-MB 157 cells (Figure 4D), which is consistent with earlier studies in which GLO1 inhibition induced apoptosis in cancer cells [42, 43]. Moreover, TLSC702 also suppressed *in vitro* tumor-sphere formation by ALDH1^high^ MDA-MB 157 and MDA-MB 468 cells (Figure 4E). The EC_50_ values of TLSC702 for tumor-sphere formation were 145.4 ± 16.6 μM (MDA-MB 157) and 67.5 ± 5.4 μM (MDA-MB 468) (Figure 4F). These results suggest that GLO1 activity is essential for cell survival and tumor formation driven by ALDH1-positive CSCs in basal-like cancers.

### Silencing GLO1 mRNA increases numbers of active caspase-3-positive and trypan blue-positive ALDH1^high^ cells

**Figure 5:**
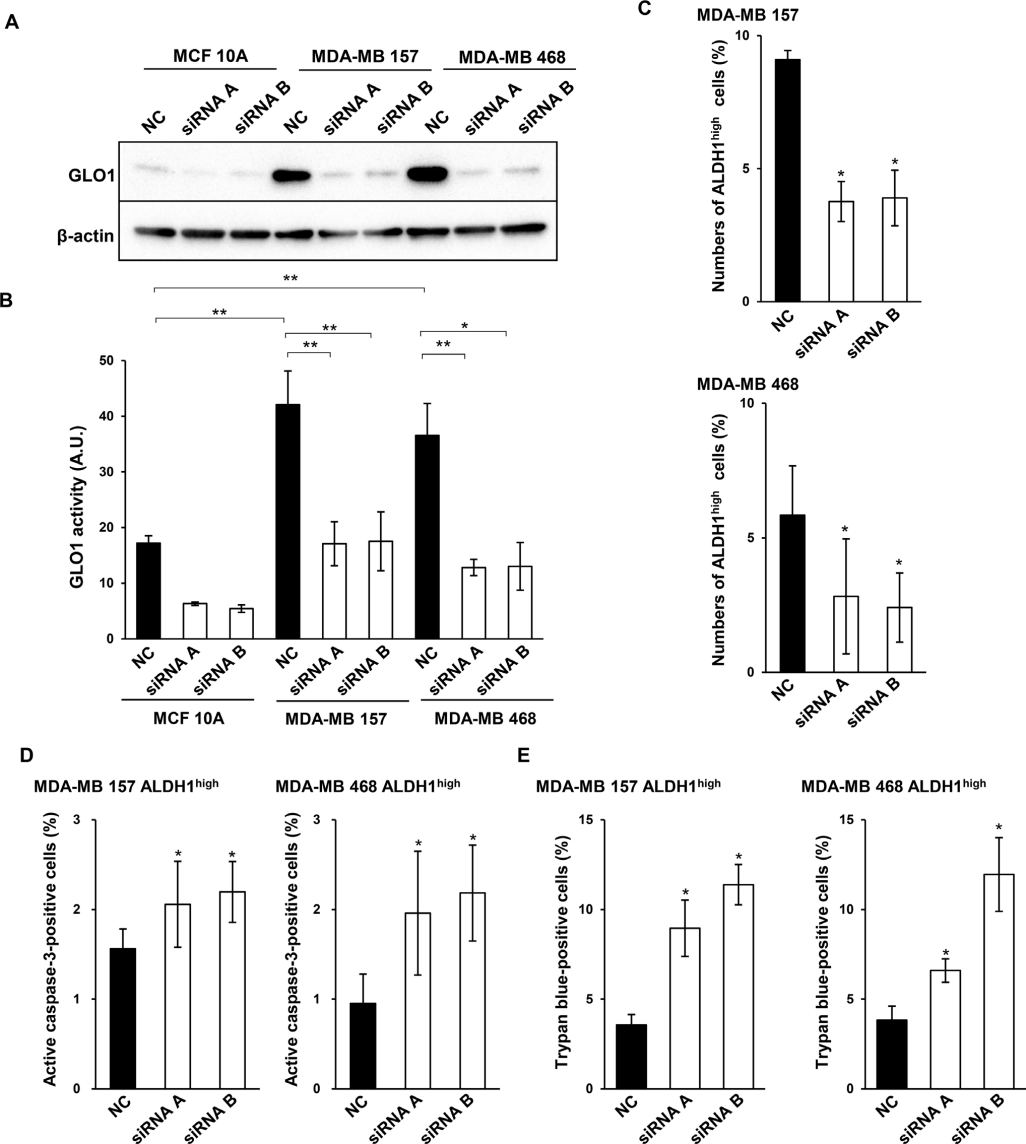
GLO1 knockdown reduces ALDH1^high^ cell numbers and induces apoptosis (**A**) Immunoblot analysis of GLO1 silenced in MCF 10A, MDA-MB 157 and MDA-MB 468 using GLO1-targeted siRNAs. β-actin was used as an internal control. (**B**) GLO1 activity measured 48 h after transfection of targeted siRNA in MCF 10A, MDA-MB 157 and MDA-MB 468 cells (1.0 × 10^4^ cells/well). GlO1 activity is expressed in arbitrary units (A.U.). ^*^p < 0.05, ^**^p < 0.01; Tukey's test. All the experiments were performed in duplicate. Data represent the mean ± SE (three independent experiments). (**C**) Numbers of ALDH1^high^ cells isolated from MDA-MB 157 cells and MDA-MB 468 cells after GLO1 knockdown were measured in ALDEFLUOR assays. ^*^p < 0.05; Students *t*-test. Data represent the mean ± SD (MDA-MB 157: three independent experiments, MDA-MB 468: four independent experiments). (**D**) Numbers of active caspase-3-positive ALDH1^high^ cells isolated from MDA-MB 157 and MDA-MB 468 cells after with GLO1 knockdown. ^*^p < 0.05; Students *t*-test. Data represent the mean ± SD (MDA-MB 157: three independent experiments, MDA-MB 468: four independent experiments). (**E**) Trypan blue staining for ALDH1^high^ cells isolated from MDA-MB 157 and MDA-MB 468 cells after GLO1 knockdown. ^*^p < 0.05; Students *t*-test. Data represent the mean ± SD (three independent experiments).

To assess the role of GLO1 in ALDH1^high^ basal-like cancer cells in detail, we used two kinds of short interfering RNAs (siRNAs) to silence GLO1 mRNA (Figure 5A). GLO1 expression and activity were lower in MCF 10A cells than MDA-MB 157 or MDA-MB 468 cells (Figure 5A and 5B). In all three cell types, GLO1 knockdown caused significant decreases in the enzymes activity (Figure 5A and 5B). Moreover, knocking down GLO1 also reduced numbers of ALDH1^high^ MDA-MB 157 and MDA-MB 468 cells (Figure 5C). These results suggest GLO1 is crucial for the viability of ALDH1-positive basal-like CSCs. Consistent with that idea, GLO1 knockdown in ALDH1^high^ MDA-MB 157 and MDA-MB 468 cells led to increases the numbers of cells positive for active caspase-3 (Figure 5D) as well as numbers of trypan blue-positive cells (Figure 5E). These results suggest that GLO1 is essential for the survival of ALDH1-positive CSCs in basal-like breast tumors.

## DISCUSSION

High levels of GLO1 mRNA and protein expression have been observed in several cancers, including breast cancer [19–33]. In the present study, we showed that high expression of *Glo1* correlates with tumor grade and the occurrence of grade 3 tumors (Figure 1C, Table 2), which is consistent with earlier immunohistochemical findings indicating that overexpression of GLO1 correlated positively with tumor grade in breast cancer [35]. In addition, *Glo1* expression was high in basal-like tumors (Figure 2A and 2B), and approximately 90% of the basal-like tumors were classified as grade 3 (Figure 2C). These results suggest GLO1 overexpression may contribute to the progression of basal-like cancers. Consistent with that idea, *Glo1* amplification contributes to the progression of gastric and liver cancers in humans [22, 25], and a *Glo1* polymorphism may influence the gene's expression in breast cancer in Egyptian women [44]. However, our study revealed that the frequency of *Glo1* amplification is low (1.0%) in breast cancer (Figure 1B) and that alteration of *Glo1* copy number, including gene amplification, did not correlate with poor clinical outcome (Supplementary Figure 1A). In addition, no genetic mutation of *Glo1* was observed. It thus appears that it is overexpression of GLO1 mRNA rather than gene amplification or mutation that contributes to disease progression in breast cancer. The molecular mechanisms underlying GLO1 overexpression without gene amplification will be an important topic to explore in the future.

Using the TCGA dataset, Kaplan-Meier analysis showed that *Glo1*^high^ was associated with a poorer prognosis (Log-rank *p* = 0.041, *n* = 447 in Supplementary Figure 1B). On the other hand, the METABRIC dataset showed no significant difference in outcomes between *Glo1*^high^ and *Glo1*^low^ (Log-rank *p* = 0.63, *n* = 1904 in Supplementary Figure 1C). In a multivariable Cox regression analysis, *Glo1*^high^ was not a factor independently predictive of overall survival among breast cancer patients in either dataset (Supplementary Table 3). These results suggest that in breast cancer, the *Glo1* expression level is not predictive of outcome.

Because we showed that high *Glo1* expression correlates with tumor grade and grade 3 tumors (Table 2 and Figure 1C), Kaplan-Meier analysis taking into consideration *Glo1* expression and neoplasm histologic grade was performed using the METABRIC dataset. In patients with grade 1 or 3 tumors, *Glo1* expression had no discernible effect on overall survival, whereas among patients with grade 2 tumors, *Glo1*^high^ was associated with a poorer prognosis (Grade 1: Log-rank *p* = 0.61, *n* = 165, Grade 2: Log-rank *p* = 0.021, *n* = 740, Grade 3: Log-rank *p* = 0.12, *n* = 927, Supplementary Figure 1D, 1E, 1F). On the other hand, in a multivariable Cox model analysis, *Glo1*^high^ was not associated with prognosis, irrespective of tumor grade (Grade 1; *p* = 0.79, Grade 2; *p* = 0.49, Grade 3; *p* = 0.15, Supplementary Table 3). Likewise, Kaplan-Meier analysis taking into consideration breast cancer subtypes showed that *Glo1*^high^ did not associate with a poorer prognosis in any subtype (Supplementary Figure 2). On the other hand, in a multivariable Cox model analysis, patients with *Glo1*^high^ luminal A or HER2-enriched cancers had poorer prognoses (Luminal A; hazard ratio 1.29, 95% CI 1.01-1.64, *p* = 0.038, *n* = 679, HER2-enriched; hazard ratio 1.62, 95% CI 1.09-2.42, *p* = 0.018, *n* = 220, Supplementary Table 4). This suggests high *Glo1* expression is a factor independently predictive of overall survival in luminal A and HER2-enriched breast cancers. Furthermore, because luminal A accounts for 49% of grade 2 tumors, the tendency toward a poorer prognosis with *Glo1*^high^ grade 2 and luminal A cancers may be related. In addition, luminal A cancers include has the highest ratio of grade 2 tumors enriched in CD44 (Figures 2C and 3A). This suggests GLO1 may be function in CD44-positive CSCs in luminal A breast cancers. GLO1 expression is reportedly high in HER2 breast cancer tissues and cell lines [45, 46] and is stimulated by HER2 signaling [46]. Therefore, the poor prognosis seen in *Glo1*^high^ HER2-enriched cancers may be related to HER2 signaling. By contrast, *Glo1*^high^ was not associated with a poorer outcome in patients with basal-like tumors (hazard ratio 0.65, 95% CI 0.40-1.05, *p* = 0.076, *n* = 199, Supplementary Table 4). However, this may reflect the smaller number of analyzed specimens.

Generally, grade 3 tumors are characterized as undifferentiated and aggressive, with loss of tubules and high mitotic activity, and are associated with the poorest clinical outcomes [36]. We confirmed that in addition to *Glo1* and *ALDH1A3*, stem cell genes such as *Notch1*, *CD133*, *cMyc*, and *HIF1A* were also highly expressed in grade 3 tumors (Figure 3A). Among breast cancer subtypes, *Notch1* and *CD133* were most highly expressed in basal-like tumors (Supplementary Figure 3). Suman et al. showed that Notch1 knockdown led to decreases in numbers of both ALDH1^+^ and ALDH1^–^ MDA-MB 231 cells [47]. In addition, Liu et al. showed that in CD133^+^ cells from TNBCs, the CSC characteristics associate with vasculogenic mimicry [48]. We therefore examined the effect of GLO1 knockdown on Notch1 and CD133 expression. As shown in Supplementary Figure 3, GLO1 knockdown did not significantly decrease levels of Notch1 or CD133 in MDA-MB 157 or MDA-MB 468 cells. This suggests GLO1 does not function in Notch1- or CD133-positive CSCs in basal-like tumors. Both c-Myc and HIF1A work as master transcriptional factors regulating glycolytic genes [49]. Recent studies have shown that glycolytic metabolism is enhanced in CSCs as compared to non-CSCs [50]. Because GLO1 is involved in detoxification of MG, its overexpression in grade 3 and basal-like tumors may be related to the enhanced glycolysis seen in ALDH1-positive CSCs.

Our results also showed that whereas *ALDH1A1* gene expression was lowest in basal-like tumors, *ALDH1A3* is highly expressed in basal-like tumors (Figure 3B). Despite a lack of correlation between *Glo1* and *ALDH1A3* expression, a major population of patients with basal-like breast cancers highly expressed both *Glo1* and *ALDH1A3* (*n* = 70 of 199, Supplementary Figure 4). In addition, basal-type cancer cell lines are more enriched with ALDH1-positive cells than are luminal type cell lines [51]. This was confirmed by Croker et al., who used specific siRNAs to show that ALDH1A3, but not ALDH1A1, contributes to ALDH1 activity in two basal-like breast cancer cell lines (MDA-MB 468 and SUM159) [52]. Our results thus strongly suggest that basal-like tumors are enriched in ALDH1-positive CSCs.

GLO1 inhibition in cancer cells leads to the accumulation of intracellular MG and induction of apoptosis [18]. TLSC702, a specific GLO1 inhibitor, induces MG accumulation and apoptosis in cancer cells [42, 43]. Our results revealed that GLO1 inhibition using TLSC702 or siRNA suppressed ALDH1^high^ cell viability and induced apoptosis (Figures 4 and 5). This suggests GLO1 is essential for survival of ALDH1-positive CSCs. In the present study, we focused on the role of GLO1 in ALDH1-positive CSCs in basal-like tumors using genomics datasets for human breast cancer. It has been reported that GLO1 knockdown suppresses proliferation and promotes apoptosis in MCF7 (luminal A), T47D (luminal A), and MDA-MB 231 (claudin-low) breast cancer cells [53]. This suggests analysis of GLO1 function in CSCs in breast cancer subtypes other than the basal-like type is needed.

Chiavarina et al. reported that Arg-pyrimidine, a MG-arginine adduct, accumulates in human breast cancer tissues to a greater degree than in non-tumoral tissues. They also found that TNBCs, which are similar to basal-like cancers, show the lowest levels of Arg-pyrimidine adducts and the highest levels of GLO1 activity among all breast cancer subtypes [33]. It appears, therefore, that basal-like breast cancers may be highly sensitive to GLO1 inhibition. Consistent with that idea, TLSC702 suppressed the viability of MDA-MB 157 and MDA-MB 468 cells at a significantly lower concentration (25-200 μM) than was required to suppress MCF 10A cells (300-600 μM) (Figure 4B). This suggests weak inhibition of GLO1 activity may be sufficient to suppress breast cancer cells, particularly if used in combination with other targeted drugs, without damaging normal cells.

Purified human ALDH1 catalyzes the conversion of MG to pyruvate [54]. Moreover, addition of MG into murine Schwann cells induces *ALDH1A3* expression [55], which confirms the likely involvement of ALDH1A3 in MG detoxification. In TNBC cells, MG also induces GLO1 expression and its activity [33]. GLO1 and ALDH1A3 may thus act cooperatively in the detoxification of MG in ALDH1-positive CSCs. Notably, in untreated GLO1-deficient murine Schwann cells, accumulation of MG is low, reflecting elevated *ALDH1A3* expression [55]. When treated with MG, however, *ALDH1A3* expression is decreased in GLO1-deficient murine Schwann cells but not in control cells. This counterintuitive response illustrates the complex relationship between GLO1, ALDH1A3 and MG and highlights the need for its further exploration in ALDH1-positive CSCs.

In our study, we observed that GLO1 activity is higher in ALDH1^high^ than ALDH1^low^ cells expressing similar levels of GLO1 (Figure 3D). This suggests GLO1 is more activated in ALDH1-positive CSCs in basal-like tumors. Ciavardelli et al. showed that glycolytic activity is higher in breast CSCs than in non-CSCs, and that inhibition of glycolysis using 2-deoxyglucose decreases proliferation and survival of breast CSCs [56]. It is well known that the activities of glycolytic enzymes are tightly regulated by their products through positive or negative feedback [57]. Analogously, GLO1 activity in ALDH1^high^ cells may be stimulated through positive feedback from MG. As mentioned, MG also induces *ALDH1A3* expression in murine Schwann cells [55], and ALDH1^high^ cells exhibit higher ALDH1A3 expression than ALDH1^low^ cells (Figure 3D). The higher GLO1 activity in ALDH1^high^ cells may thus reflect MG-dependent positive feedback in ALDH1-positive CSCs survival.

We observed that in addition to breast cancer, *Glo1* expression is also higher colon and ovarian cancers than in normal tissues (Supplementary Figure 5). ALDH1A3 is also the primary ALDH1 isoform in human colon cell lines [58], and ALDH1A3 is highly expressed in ovarian cancer tissues [59]. This suggests it will be important to investigate the roles of GLO1 in ALDH1-positive CSCs in these cancers as well.

## MATERIALS AND METHODS

### Analysis of the TCGA dataset

The Cancer Genome Atlas (TCGA) breast cancer dataset [60] was downloaded from Oncomine (https://www.oncomine.org, Compendia Bioscience, Ann Arbor, MI, USA) [61]. GLO1 mRNA expression was compared between normal and cancer tissues, both of which were available from TCGA breast cancer dataset. Levels of GLO1 mRNA expression (reporter: A_32_P53822) were displayed using log2 median-centered ratio boxplots for normal tissue vs. cancer tissue. The *p* values were calculated using the Mann-Whitney *U* test. The clinical data from breast cancer patients are summarized in Supplementary Table 1. The median age at the time of diagnosis was 57.9 years (aged 26 to 90 years). The dataset contains mRNA expression data from 61 normal breast tissue samples and 532 primary breast tumor samples. We defined two groups based on *Glo1* expression: *Glo1*^high^ (log2 median-centered intensity > 0) and *Glo1*^low^ (log2 median-centered intensity < 0) in Table 1. The *p* values for the correlations between the clinicopathological data and *Glo1* expression were calculated using the χ^2^ test.

### Analysis of the METABRIC dataset

The Molecular Taxonomy of Breast Cancer International Consortium (METABRIC) dataset [62, 63] was downloaded from cBioPortal (http://www.cbioportal.org/) [64, 65]. Analyses of gene expression, gene amplification, genetic mutation, and clinicopathological data were performed as previously described [39]. The clinicopathological data from the breast cancer patients are summarized in Supplementary Table 2. These clinical data, including the diagnostic criteria, were downloaded from cBioPortal. Neoplasm histologic grade (Tumor grade) is a numeric value expressing the degree of abnormality of cancer cells and is an index of differentiation and aggressiveness [36]. The median age at the time of diagnosis was 61.1 years (aged 21 to 96 years). Quantitative variables were analyzed using the Kruskal-Wallis test with the Steel-Dwass test in Figures 1C and 3B. Values of *p* < 0.05 were considered significant. We defined two groups based on *Glo1* expression: *Glo1*^high^ (z-score > 0) and *Glo1*^low^ (z-score < 0) in Table 2. The *p* values for the correlations between the clinicopathological data and the *Glo1* expression were calculated using the χ^2^ test. For the analysis of stemness genes expression (z-score) in Figure 3A, the average value of stemness gene expression in each tumor grade was calculated and drawn as a Heatmap using R version 3.4.1 (R Foundation for Statistical Computing, Vienna, Austria).

### PAM50 subtyping

Breast cancers are classified based on the 50 gene prediction analysis of microarray (PAM50) subtype predictor, which classifies tumors as normal-like, luminal A, luminal B, HER2-enriched, claudin-low and basal-like. Because the TCGA dataset from Oncomine does not include the PAM50 classification, we combined the TCGA datasets from Oncomine and cBioportal based on patient ID. Samples without PAM50 data were excluded from the analysis. We then analyzed and compared clinical specimens of normal vs. patients with each cancer subtype in Figure 2A. The *p* values were calculated using the Wilcoxon signed rank test.

### Cell culture

Human basal-like breast cancer cell lines (MDA-MB 157 and MDA-MB 468) and a human normal-like (non-transformed) mammary epithelial cell line (MCF 10A) were obtained from the American Type Culture Collection (ATCC, Manassas, VA, USA). MCF 10A cells were grown in mammary epithelial cell growth medium (MEGM; Lonza) according to instructions from the ATCC and were used in the experiments for immunoblotting and assessing GLO1 activity. WST-8 assays with MCF 10A cells were carried out in MEGM supplemented with 10% FBS. The cancer cell lines were cultured as previously described [39].

### Chemicals and reagents

3-(1,3-Benzothiazol-2-yl)-4-(4-methoxyphenyl) but-3-enoic acid (TLSC702) was purchased from Namiki Shoji Co., Ltd. (Japan) and dissolved in DMSO. Mouse anti-β-actin monoclonal antibody, mouse anti-GLO1 monoclonal antibody and rabbit anti-ALDH1A3 polyclonal antibody were purchased from Proteintech Group, Inc. (U.S.A.), Santa Cruz Biotechnology (U.S.A.) and Invitrogen (U.S.A.), respectively. Horseradish peroxidase (HRP)-conjugated goat anti-mouse IgG and anti-rabbit IgG were purchased from Cell Signaling Technology (U.S.A.).

### Immunoblotting

Immunoblotting was performed as previously described [39]. Briefly, proteins were separated on SDS-PAGE (8% or 12% gel) and transferred Immobilon-P membranes (Millipore, ISEQ00010). The membranes were then blocked with 5% skim milk in TBST, incubated with the primary antibodies, and probed using horseradish peroxidase-conjugated secondary antibodies. Specific signals were detected using the chemiluminescence reagents Immunostar Basic (Wako), Immunostar LD (Wako) or EzWestLumiOne (ATTO) with ChemiDoc MP (BIO RAD).

### ALDEFLUOR assay

ALDH1^high^ cells were isolated from MDA-MB 157 and MDA-MB 468 cells using an ALDEFLUOR assay kit (Stem Cell Technology) according to the manufacturer's instructions. As a negative control for the ALDEFLUOR assay, cells were incubated with the ALDH1 inhibitor diethylaminobenzaldehyde (DEAB). Approximately 5–10% of the total ALDH1^high^ cells were sorted by the cell sorter (FACS Aria II, BD Bioscience), taking the negative control into consideration. ALDH1^low^ cells were sorted from the same proportion as ALDH1^high^ cells using the lowest ALDH1 activity population.

### *In vitro* GLO1 assay

GLO1 activity was measured *in vitro* as previously described [66]. Cells (Figure 3D: 6.0 × 10^3^/well, Figure 5B: 1.0 × 10^4^/well) were seeded into 96-well plates (Thermo 161093). After 24 h (Figure 3D) or after 48 h of siRNA transfection (Figure 5B), the cells were lysed in assay mixture containing 0.2% Triton X-100 and 0.3% NP-40. The GLO1 activity in the lysate was detected spectrophotometrically by monitoring the increase in absorbance at 240 nm due to formation of S-D-lactoylglutathione for 5 min at 25° C. The standard assay mixture contained 7.9 mM MG, 1 mM glutathione, 14.6 mM magnesium sulfate, and 182 mM imidazole-HCl, pH 7.0. Before initiating the reaction by adding cell lysate, the assay mixture was allowed to stand for 15 min to ensure equilibration of hemithioacetal formation.

### WST-8 assay

Unsorted MCF 10A, MDA-MB 157 and MDA-MB 468 cells (Figure 4B: 5.0 × 10^3^/well) as well as ALDH1^high^ cells derived from MDA-MB 157 and MDA-MB 468 cells (Figure 4C: 1.0 × 10^3^/well) were seeded into 96-well plates (Sigma) and incubated for 24 h. TLSC702 was then added to the cultures, and the cells were incubated for an additional 3 days, after which cell viability was assessed using WST-8 assays (Cell Counting Kit-8 (DOJINDO)). The formazan dye formed was measured using an ARVO™ MX (PerkinElmer) or Sunrise Remote (TECAN) at 450 nm. Numerical values of the test groups are expressed relative to the 0.6% DMSO-treated group.

### Tumor-sphere culture

Tumor-spheres were cultured as previously described [39]. ALDH1^high^ cells (1 × 10^3^/well) were cultured in ultralow attachment 96-well plates (Greiner) and treated with TLSC702 for 6 days. CellTiter-Glo^®^ luminescence assays (Promega) were performed with a TR717 Micro plate Luminometer (TROPIX) using a 96-well Micro-assay-plate (Greiner). Numerical values for the test groups are expressed to the 0.6% DMSO-treated group.

### Caspase-3/7 fluorometric assay

Caspase-3/7 activities were assayed using the Apo-ONE™ Homogeneous Caspase-3/7 assay (Promega G7790) according to the manufacturer's instructions. ALDH1^high^ cells (1 × 10^3^/well) were seeded into black 96-well fluorescence culture plates (Greiner bio-one 655090) and incubated for 24 h, after which they were treated with TLSC702 for an additional 5 days. Equal volumes of DMEM and Apo-ONE™ caspase reagent (1:100 profluorescent substrate and lysis buffer) were then added to cells, and the mixture was incubated for 30 min. Fluorescence (excitation, 485 nm; emission, 512 nm) was measured using a fluorescence plate reader (SPECTRA max GEMINI XPS [Molecular Devices]). Background fluorescence was determined as the fluorescence from DMEM alone and deducted from all experimental values.

### siRNA transfection

siRNA oligonucleotides were transfected at a final concentration of 20 nM using Lipofectamine RNAiMAX transfection reagents (Invitrogen) according to manufacturer's instructions. The following siRNAs were used: NC siRNA, MISSION siRNA Universal Negative Control (Sigma-Aldrich), GLO1 siRNA A (GUGAUUCAAGAUAUUUACATT; Sigma-Aldrich), and GLO1 siRNA B (AGAAGCAUCUAGGACUGAUTT; Bioneer).

### Detection of apoptotic cells

Following siRNA transfection, the cells were cultured for 48 h. ALDH1^high^ cells were then isolated from the siRNA transfectants and plated. For immunofluorescent staining, ALDH1^high^ cells cultured for 24 h on Lab-Tek chamber slides (Nalge Nunc International, Rochester, NY, USA) were fixed with 2% paraformaldehyde and permeabilized with 0.1% Triton X-100. Immunofluorescent staining was performed using anti-cleaved caspase-3 antibody (1:500; CST #9661). Alexa 488-conjugated goat anti-rabbit antibody (Invitrogen) was used as the secondary antibody. Hoechst 33342 (Invitrogen) was used for nuclear staining. The slides were mounted with ProLong Gold antifade reagent (Invitrogen). For trypan blue staining, ALDH1^high^ cells were cultured for 24 h in 12-well culture plates (3.0 × 10^4^ cells/well) (Thermo Scientific). After staining with 0.4 w/v% trypan blue solution (Wako 207-17081), the cells were counted manually.

## CONCLUSIONS

In the present study, we used human breast cancer genomics dataset analysis to show that GLO1 expression is elevated in human basal-like breast cancer tissues, and that inhibition of GLO1 suppresses cell viability and tumor-sphere formation by ALDH1^high^ cells. These findings suggest GLO1 is essential for cell viability and for tumor formation by ALDH1-positive CSCs. As such, GLO1 is a potential therapeutic target for treatment ALDH1-positive CSCs in basal like breast cancers.

## SUPPLEMENTARY MATERIALS FIGURES AND TABLES


