# Supplementary material for: Glyoxalase 1 gene is highly expressed in basal-like human breast cancers and contributes to survival of ALDH1-positive breast cancer stem cells
Source: Oncotarget. 2018 Nov 23;9(92):36515–29. doi: 10.18632/oncotarget.26369 (PMC6284866; doi:10.18632/oncotarget.26369)
Supplement: Supplementary file 1 [file oncotarget-09-36515-s001.pdf]

Glyoxalase 1 gene is highly expressed in basal-like human breast cancers and contributes to survival of ALDH1-positive breast cancer stem cells

SUPPLEMENTARY MATERIALS

Chemicals and reagents

Rabbit anti-Notch1 polyclonal antibody (sc-6014) and APC-conjugated anti-CD133 antibody (130090854) were purchased from Santa Cruz Biotechnology (U.S.A.) and Miltenyi Biotec (U.S.A.), respectively.

Flow cytometric analysis

After transfection of siRNAs for 48 h, cells (2.0×10^6 cells/mL) were dissociated in FACS buffer (1 × PBS supplemented with 2% FBS), incubated with APC-conjugated anti-CD133 antibody for 10 min on ice, and analyzed using a FACS Calibur flow cytometer (BD Biosciences). Unstained cells were used as a gating control. The data was analyzed using FlowJo 8.8.4 software. Three independent assays were performed for confirmation.

Survival analysis

From the TCGA dataset in Oncomine, we obtained overall survival follow-up times (days) and survival statuses for 447 of 532 patients. We defined two groups based on *Glo1* expression: $Glo1^{high}$ (log2 median centered intensity > 0) and $Glo1^{low}$ (log2 median centered intensity < 0) (Supplementary Figure 1A). From the METABRIC dataset in cBioportal, we obtained the overall survival (months) and overall survival status for 1904 patients. We defined another two groups based on *Glo1* expression: $Glo1^{high}$ (z-score > 0) and $Glo1^{low}$ (z-score < 0) (Supplementary Figure 1B). Differences between overall survival in Kaplan-Meier analyses were determined using the log-rank test. Multivariable Cox regression was used to evaluate the influence of *Glo1* expression with age and tumor stage as confounding factors. All statistical analyses were performed using R version 3.4.1 (R Foundation for Statistical Computing, Vienna, Austria).

Analysis of TCGA dataset of several cancers

The Cancer Genome Atlas (TCGA) dataset was downloaded from Oncomine (https://www.oncomine.org, Compendia Bioscience, Ann Arbor, MI, USA). Levels of GLO1 mRNA expression in colon, lung, renal, uterine (reporter: A_32_P53822), brain, and ovarian cancer and leukemia (reporter: 200681) tissues and the corresponding normal tissues were displayed using log2 median-centered ratio boxplots for normal vs. cancer tissue. The p values are calculated using the Mann-Whitney U test.

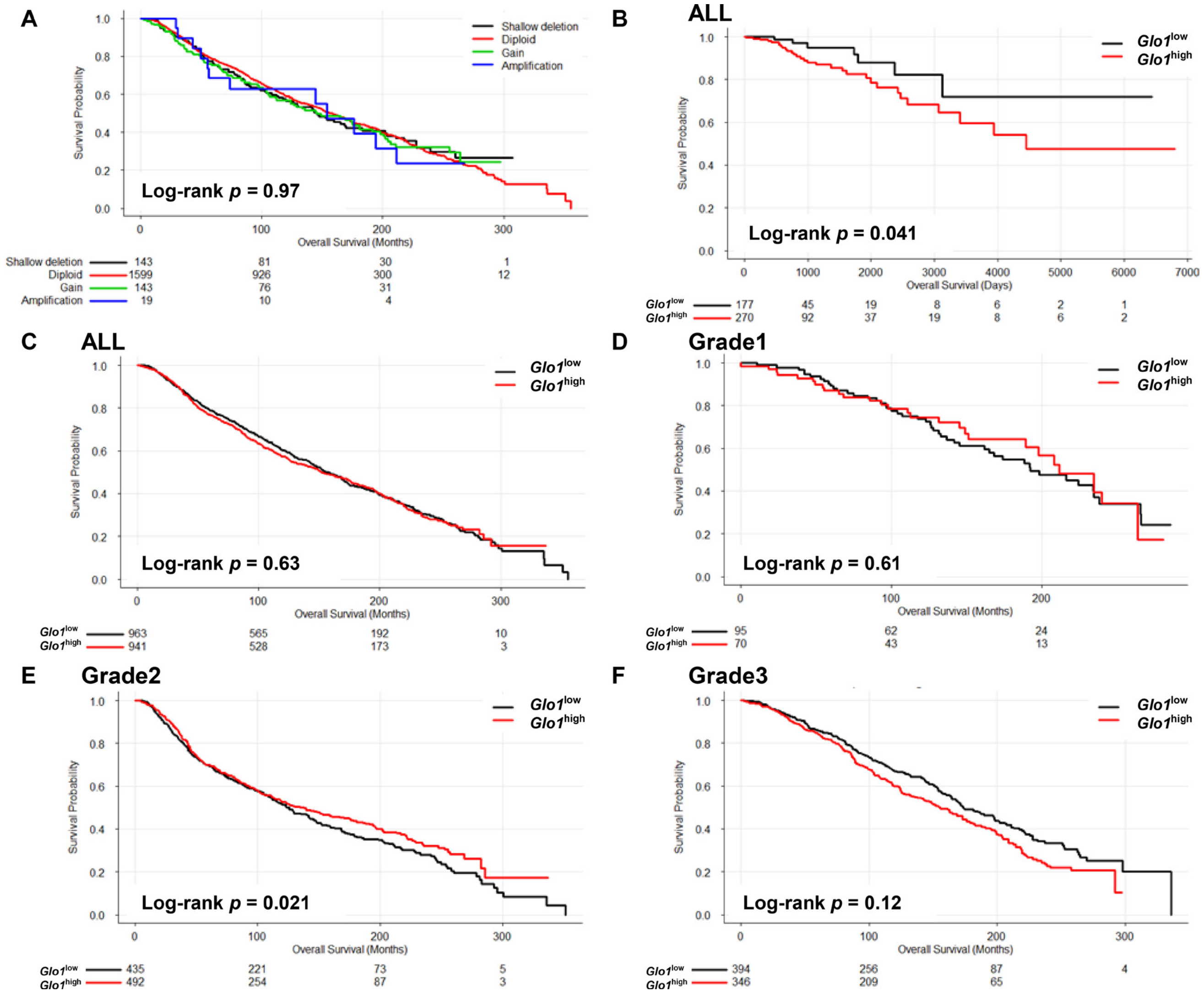

Supplementary Figure 1: Effect of *Glo1* expression on overall survival in breast cancer. (**A**) Kaplan–Meier analysis of overall survival of breast cancer patients, taking into consideration *Glo1* copy number alteration status, including shallow deletion (n = 143), diploid (n = 1599), gain (n = 143), and amplification (n = 19) (from the METABRIC dataset). (**B**) Kaplan-Meier analysis of overall survival of breast cancer patients with *Glo1*^{high} (n = 270) and *Glo1*^{low} (n = 177) (from the TCGA dataset). (**C**) Kaplan-Meier analysis of overall survival of breast cancer patients with *Glo1*^{high} (n = 941) and *Glo1*^{low} (n = 963) (from METABRIC dataset). (**D–F**) Kaplan–Meier analysis of overall survival of breast cancer patients, taking into consideration *Glo1* expression level and tumor grade (D); *Glo1*^{high} (n = 69) vs. *Glo1*^{low} (n = 96) in Grade 1, (E); *Glo1*^{high} (n = 346) vs. *Glo1*^{low} (n = 394) in Grade 2, (F); *Glo1*^{high} (n = 492) vs. *Glo1*^{low} (n = 435) in Grade 3) (from METABRIC dataset).

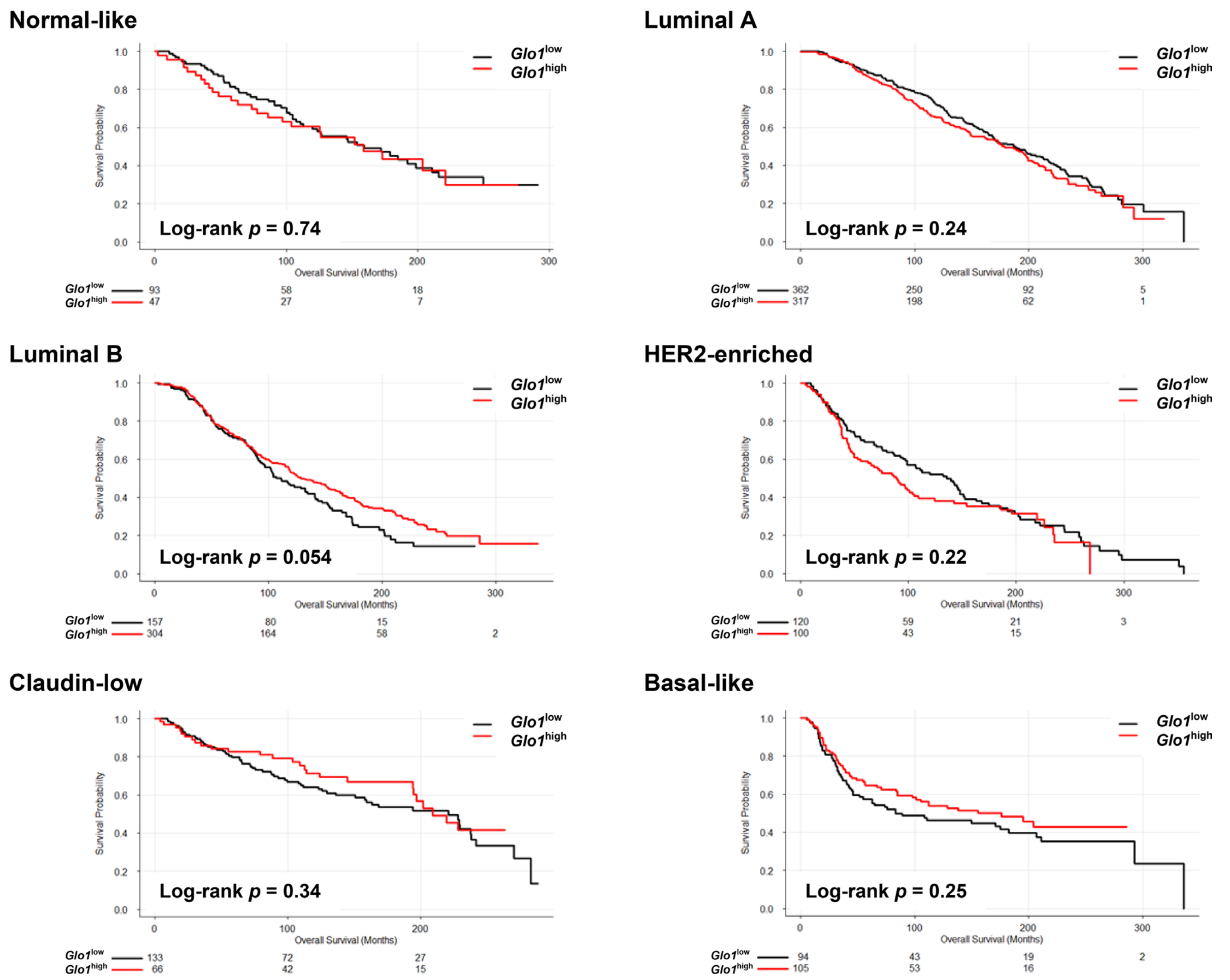

Supplementary Figure 2: Effect of *Glo1* expression on overall survival in breast cancer subtypes. Kaplan–Meier analysis showing the effect of *Glo1* expression on overall survival of patients with the indicated breast cancer subtype (Upper: left, $Glo1^{high}$ ($n = 47$) vs. $Glo1^{low}$ ($n = 93$) in Normal-like; right, $Glo1^{high}$ ($n = 317$) vs. $Glo1^{low}$ ($n = 362$) in Luminal A; Middle:left, $Glo1^{high}$ ($n = 304$) vs. $Glo1^{low}$ ($n = 157$) in Luminal B. right, $Glo1^{high}$ ($n = 100$) vs. $Glo1^{low}$ ($n = 120$) in HER2-enriched; lower:left, $Glo1^{high}$ ($n = 66$) vs. $Glo1^{low}$ ($n = 133$) in Claudin-low; right, $Glo1^{high}$ ($n = 105$) vs. $Glo1^{low}$ ($n = 94$) in Basal-like) (from the METABRIC dataset).

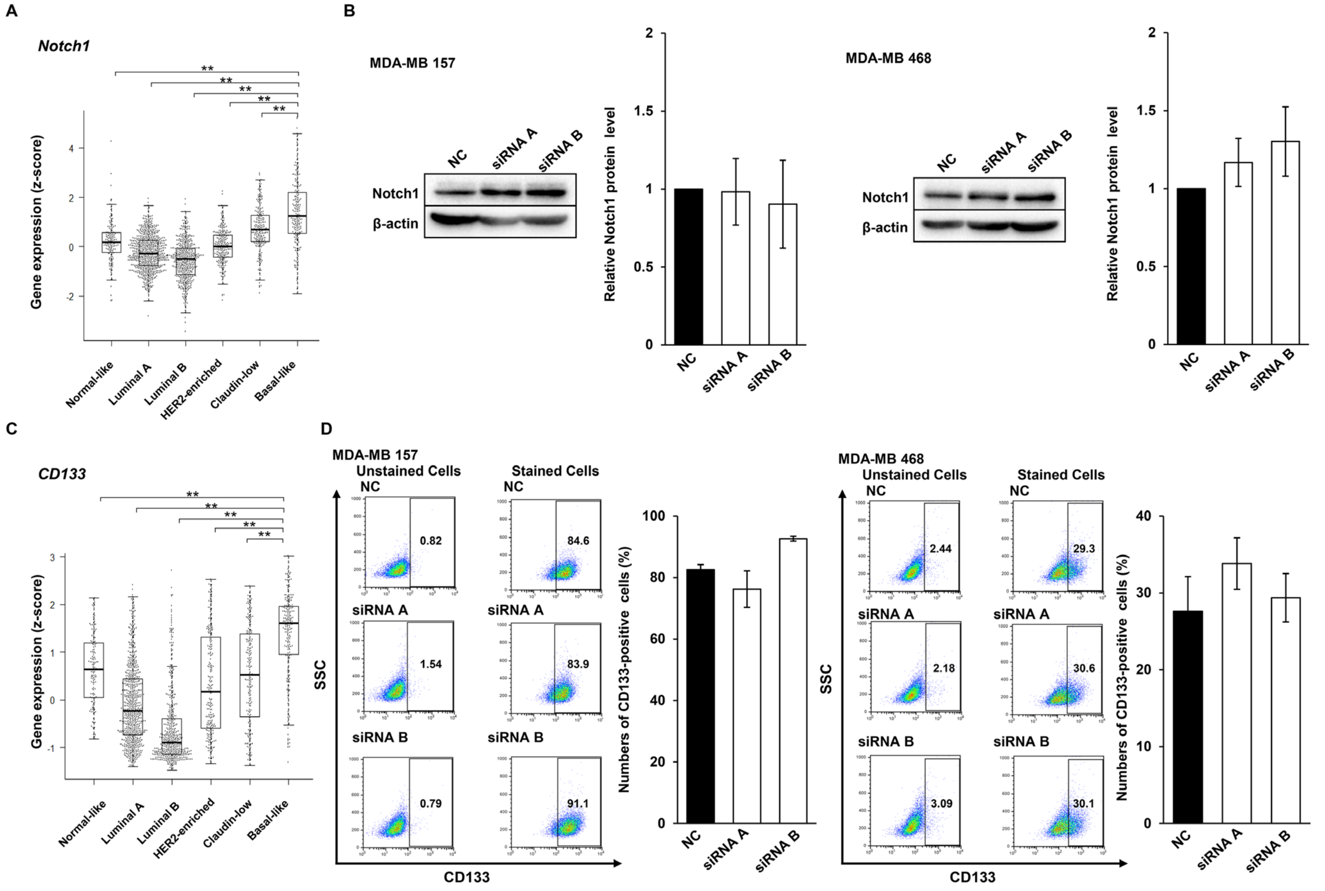

Supplementary Figure 3: Notch1 and CD133 levels after GLO1 knockdown. (**A**) *NOTCH1* gene expression in breast cancer subtypes (from METABRIC dataset): center line, median; box limits, upper and lower quartiles; whiskers, ± 1.5 × interquartile range (IQR); points, all data points. $^{**}p < 0.01$; Kruskal-Wallis test with Steel-Dwass test. (**B**) Immunoblot analysis of Notch1 expression in MDA-MB 157 and MDA-MB 468 cells after silencing *Glo1* using targeted siRNA. (**C**) *CD133* gene expression in breast cancer subtypes (from METABRIC dataset): center line, median; box limits, upper and lower quartiles; whiskers, ± 1.5 × interquartile range (IQR); points, all data points. $^{**}p < 0.01$; Kruskal-Wallis test with Steel-Dwass test. (**D**) Numbers of CD133-positive cells isolated from MDA-MB 157 cells and MDA-MB 468 cells after GLO1 knockdown (Left, representative FACS pattern; Right, Quantitative values). $^{*}p < 0.05$; Students *t*-test. Data represent the mean ± SD (three independent experiments each).

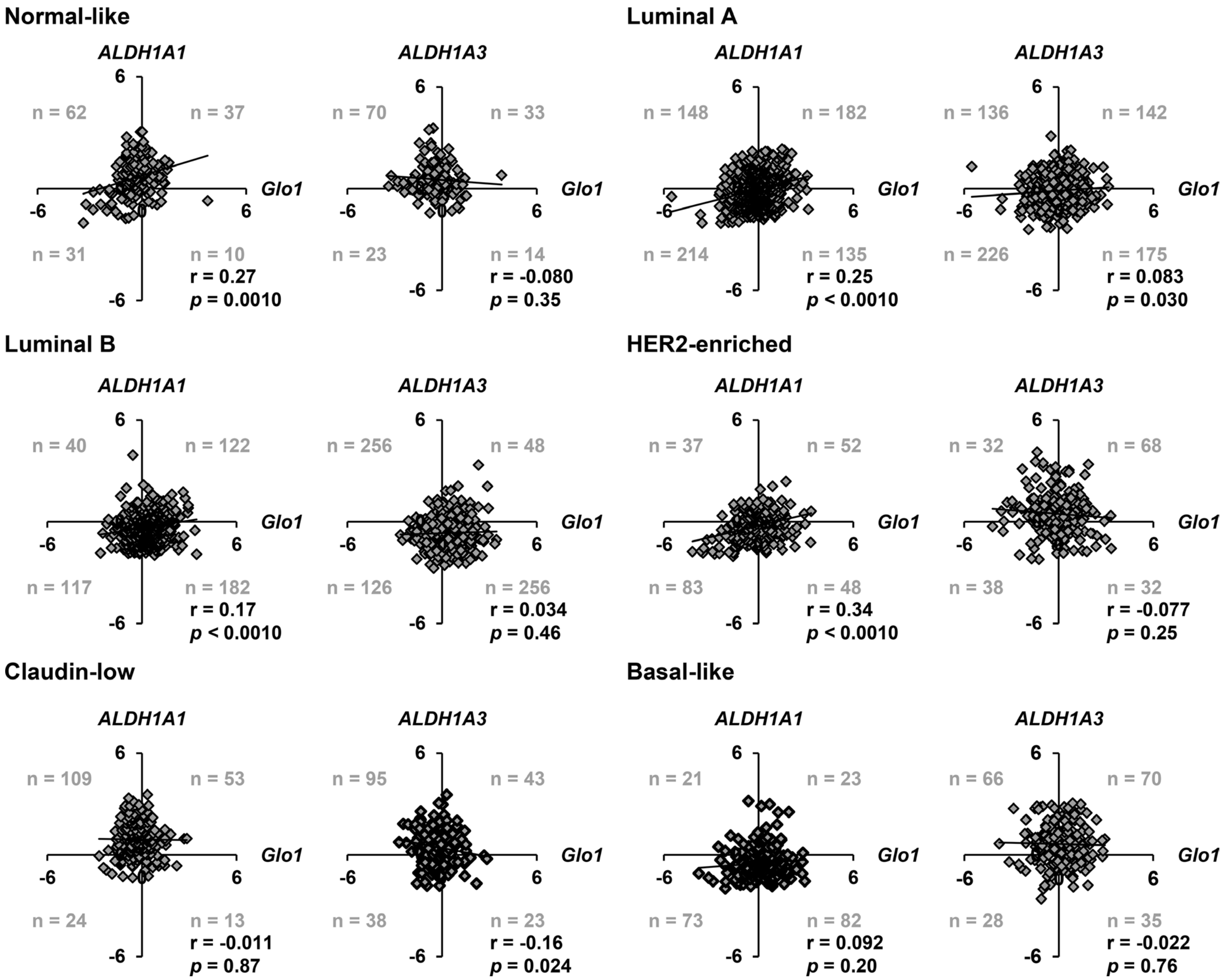

Supplementary Figure 4: Correlation of *Glo1* expression with expression of *ALDH1A1* and *ALDH1A3* in the indicated breast cancer subtypes. Values are shown as scattered plots. Pearson's correlation coefficients (r) and *p* values are indicated.

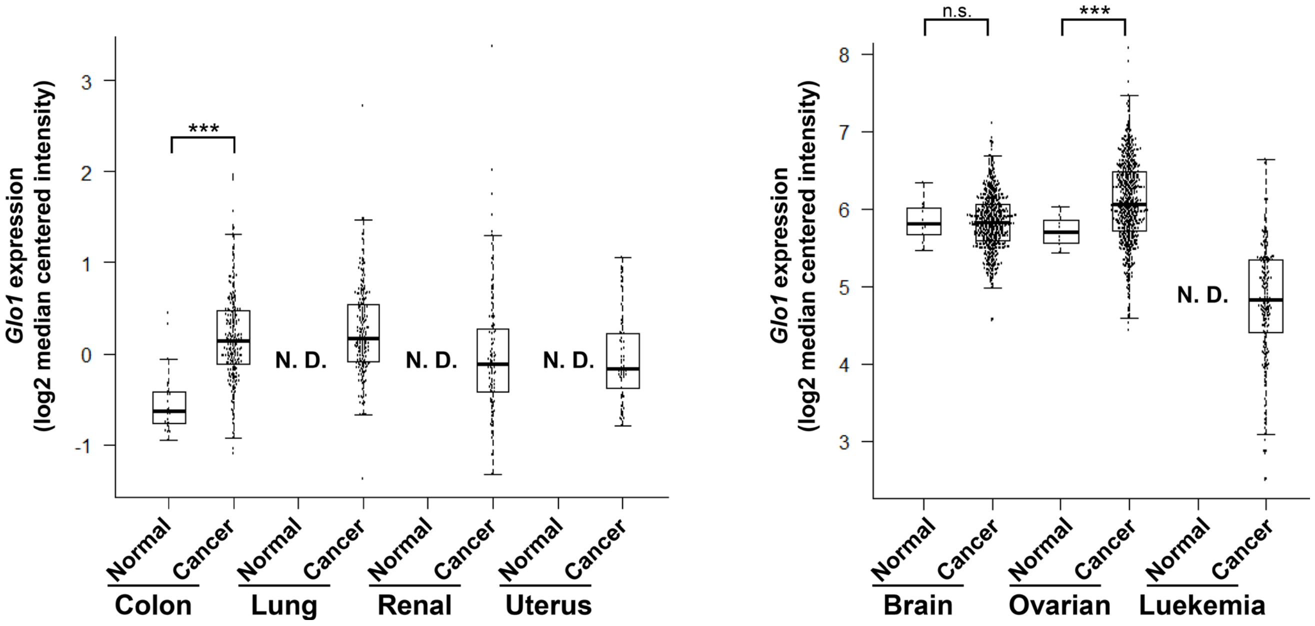

Supplementary Figure 5: Comparison *Glo1* expression between normal tissue and the indicated cancers. Box plots compare *Glo1* expression in each normal tissue and corresponding cancer tissue (from the TCGA dataset) (N. D. = no data): center line, median; box limits, upper and lower quartiles; whiskers, ± 1.5 × interquartile range (IQR); points, all data points (left, reporter A_32_P53822; right, reporter 200681). $^{***}p < 0.001$, n.s. = not significant; Mann–Whitney *U* test.

Supplementary Table 1: TCGA clinicopathological data from oncomine

Variable		n = 593	(%)
Age	57.9 ± 13.1 (26-90)		
	>61	206	(34.7)
	≤61	279	(47.0)
	Not informative	108	(18.2)
Gender	Male	3	(0.5)
	Female	484	(81.6)
	Not informative	106	(17.9)
Cancer and normal type	Breast	61	(10.3)
	Tumor	532	(89.7)
**Tumor histological type**		**n = 532**	**(%)**
Estrogen receptor status	Positive	273	(51.3)
	Negative	95	(17.9)
	Not informative	164	(30.8)
Progesterone receptor status	Positive	228	(42.9)
	Negative	144	(27.1)
	Not informative	160	(43.4)
ERBB2 status	Positive	73	(13.7)
	Negative	228	(42.9)
	Not informative	231	(43.4)
Tumor stage	Stage I	49	(9.2)
	Stage II	243	(45.7)
	Stage III	93	(17.5)
	Stage IV	13	(2.4)
	Not informative	134	(25.2)
Tumor histologic subtype	Intraductal Cribriform Breast Adenocarcinoma	3	(0.6)
	Invasive Breast Carcinoma	76	(14.3)
	Invasive Ductal Breast Carcinoma	392	(73.7)
	Invasive Lobular Breast Carcinoma	36	(6.8)
	Male Breast Carcinoma	3	(0.6)
	Mixed Lobular and Ductal Breast Carcinoma	7	(1.3)
	Mucinous Breast Carcinoma	4	(0.8)
	Not informative	11	(2.1)

Supplementary Table 2: METABRIC clinicopathological data from cBioportal

Variable		*n* = 1904	(%)
Age	61.1 ± 13.0 (21.9–96.3)		
	>61	993	(52.2)
	≤61	911	(47.8)
Gender	Male	0	0.0
	Female	1904	(100.0)
Tumor size (mm)	≥5 cm	142	(7.5)
	<5 cm	1744	(91.6)
	Not informative	18	(0.9)
Histological type			
ER	+	1459	(76.6)
	–	445	(23.4)
PgR	+	1009	(53.0)
	–	895	(47.0)
HER2	+	236	(12.4)
	–	1668	(87.6)
Tumor stage	Stage 0	4	(0.2)
	Stage I	475	(24.9)
	Stage II	800	(42.0)
	Stage III	115	(6.0)
	Stage IV	9	(0.5)
	Not informative	501	(26.3)
Neoplasm histologic grade	Grade 1	165	(8.7)
	Grade 2	740	(38.9)
	Grade 3	927	(48.7)
	Not informative	72	(3.8)
Pam50 + claudin-low subtype	Normal-like	140	(7.4)
	Luminal A	679	(35.7)
	Luminal B	461	(24.2)
	HER2-enriched	220	(11.6)
	Claudin-low	199	(10.5)
	Basal-like	199	(10.5)
	Not informative	6	(0.3)
Tumor other histologic subtype	DCIS	2	(0.1)
	IDC	1727	(90.7)
	ILC	141	(7.4)
	BENIGN	1	(0.1)
	Invasive Tumor	9	(0.5)
	Mixed Nst and a special type	3	(0.2)
	Other	10	(0.5)
	Other Invasive	9	(0.5)
	Not Classified	2	(0.1)

Supplementary Table 3: Effect of *Glo1* expression on relative overall survival probability determined by multivariate analysis in breast cancer

**Cox proportional regression hazards model**				
			**Multivariate analysis**	
		Hazard ratio	95% confidence interval	*p* value
TCGA	ALL	2.28	0.84–6.22	0.11
METABRIC	ALL	0.99	0.86–1.14	0.91
	Grade 1	1.08	0.59–1.99	0.79
	Grade 2	1.09	0.86–1.38	0.49
	Grade 3	0.87	0.72–1.05	0.15

Supplementary Table 4: Effect of *Glo1* expression on relative overall survival probability determined by multivariate analysis in breast cancer subtypes

**Cox proportional regression hazards model**			
		**Multivariate analysis**	
	Hazard ratio	95% confidence interval	*p* value
Normal-like	1.14	0.60–2.18	0.68
Luminal A	1.29	1.01–1.64	0.038
Luminal B	0.76	0.57–1.01	0.062
HER2-enriched	1.62	1.09–2.42	0.018
Claudin-low	0.64	0.37–1.09	0.10
Basal-like	0.65	0.40–1.05	0.076
